# The Elusive Evidence of Volcanic Lightning

**DOI:** 10.1038/s41598-017-15643-8

**Published:** 2017-11-14

**Authors:** K. Genareau, P. Gharghabi, J. Gafford, M. Mazzola

**Affiliations:** 1 Department of Geological Sciences, University of Alabama, Box 870338, Tuscaloosa, Alabama 35487 USA; 2 Department of Electrical and Computer Engineering, Mississippi State University, Starkville, Mississippi 39762 USA; 3 Center for Advanced Vehicular Systems at Mississippi State University, Starkville, Mississippi 39759 USA

## Abstract

Lightning strikes are known to morphologically alter and chemically reduce geologic formations and deposits, forming fulgurites. A similar process occurs as the result of volcanic lightning discharge, when airborne volcanic ash is transformed into lightning-induced volcanic spherules (LIVS). Here, we adapt the calculations used in previous studies of lightning-induced damage to infrastructure materials to determine the effects on pseudo-ash samples of simplified composition. Using laboratory high-current impulse experiments, this research shows that within the lightning discharge channel there is an ideal melting zone that represents roughly 10% or less of the total channel radius at which temperatures are sufficient to melt the ash, regardless of peak current. The melted ash is simultaneously expelled from the channel by the heated, expanding air, permitting particles to cool during atmospheric transport before coming to rest in ash fall deposits. The limited size of this ideal melting zone explains the low number of LIVS typically observed in volcanic ash despite the frequent occurrence of lightning during explosive eruptions.

## Introduction

Volcanic ash produced during explosive eruptions with documented lightning contain rounded, glassy particles due to melting and re-solidification of the material into lightning-induced volcanic spherules (LIVS)^1^. Although LIVS have been observed in ash fall deposits from several volcanoes (Pavlof, Redoubt, and Okmok, U.S.A.; Sakurajima, Japan; Eyjafjallajökull, Iceland), they represent a very low percentage of the total ash grain population, even though volcanic lightning is a common phenomenon during explosive eruptions^2–5^. The existence of fulgurites, glassy products formed in rocks and sediments struck by cloud-to-ground (CG) lightning, provide direct evidence that geologic materials can be melted due to natural high-current discharge^6–8^. Temperatures within the lightning discharge channel can reach ~30000 °C^9–15^, which is over an order of magnitude higher than the melting point of igneous minerals and glasses. Numerous analyses of fulgurites have shown that, in most cases, the original country rock is chemically altered and high-temperature mineral phases and/or amorphous glasses are produced^16–20^. Some previous analyses of fulgurites found throughout the world have revealed that the geologic materials are chemically reduced as the result of lightning discharge^16,20,21^. This potential for morphological and chemical change implies that the ordered structure and composition of volcanic ash exposed to lightning discharge will be fundamentally altered. Despite the evidence of volcanic lightning in the geologic record provided by LIVS^1^, and changes in ash particle morphology induced by high-voltage insulator flashover experiments^22^, the extent of ash transformation in relation to location within the discharge channel has not been constrained until now.

**Figure 1:**
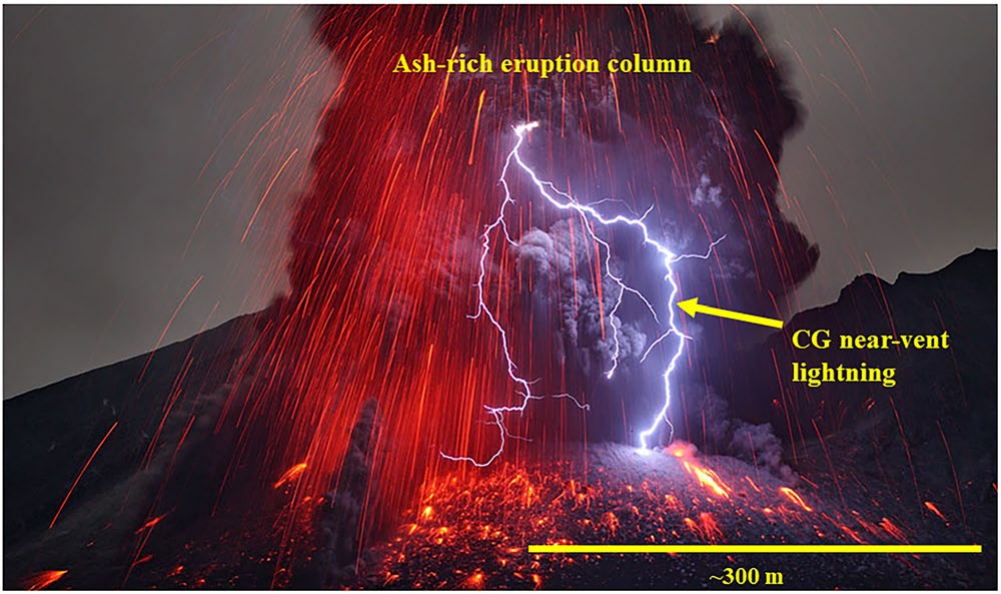
Volcanic lightning discharge. Near-vent lightning discharge during an explosive eruption at Sakurajima volcano in Japan, showing a cloud-to-ground (CG) return stroke between the crater rim and the ash-laden eruption column (photo by Martin Rietze).

Classification of volcanic lightning “types” (vent, near-vent, plume lightning) is based upon the length and timescale of discharge in addition to distance from the vent^23,24^. Observations of volcanic lightning reveal that near-vent discharges will range from tens to hundreds of meters in length and are on the order of 1 to 2 m in diameter^25^ (Fig. 1), while plume lightning at higher altitudes, downwind from the vent, may reach kilometers in length^5^. These observed diameters include the central conductive core of the channel and the surrounding non-conductive coronal sheath^26,27^. Explosive volcanic eruptions display both CG flashes and intra-cloud flashes in the near-vent region^25,28^ (Fig. 1), with the former generally lasting longer in duration. An examination of CG lightning at Sakurajima volcano (Japan) revealed peak currents (*I*_*max*_) of 2 kA, but these analyses focused on only near-vent discharges and did not include larger plume lightning discharges^28^. Studies of thunderstorm lightning have occurred for decades, with many of these efforts able to determine the *I*_*max*_ of CG return strokes using various methods^11,29–33^. Although values can substantially vary, the median *I*_*max*_ of these return strokes is roughly 30 kA, with only 5% reaching *I*_*max*_ values greater than 100 kA^32^. Based upon previous studies of thunderstorm CG return strokes, and the limited analyses of volcanic discharges, lightning during explosive eruptions can potentially span a wide range of *I*_*max*_ values (2 kA-100 kA) depending on type and size. The experiments conducted here utilise current impulses to simulate a CG return stroke at the higher end (~100 kA) of the potential *I*_*max*_ range for volcanic lightning.

Volcanic lightning occurs in regions of the eruptive column and plume that have widely variable ash particle concentrations, ranging from 10^4^ to 10^8^ particles per m^3^ for the grain sizes (1–10 μm)^34,35^ utilised in the experiments presented here. This would suggest that millions of ash particles should be morphologically transformed by the occurrence of lightning discharge, but numerical models indicate that both the timescale of lightning discharge and the size of exposed ash particles determine the likelihood of forming LIVS^36^. Observations of natural ash falls from several volcanoes where lightning has been documented reveal very few LIVS, typically comprising 1% or less of grains^1^. Thus, the study presented here addresses the location within the discharge channel where volcanic ash will be affected and the resulting proportion of volcanic ash within an explosive eruption column that will be morphologically altered when subjected to lightning discharge, revealing why textural evidence of volcanic lightning is scarce in ash fall deposits.

Due to the high voltage, high current, and high temperature of lightning, effects on exposed materials are damaging. Modern aircraft are particularly susceptible to lightning damage due to construction using low-weight polymer composite materials, which cannot effectively dissipate electrical current compared to traditional aluminum^37^. Several studies have attempted to constrain the extent of thermo-mechanical damage to key components of aircraft struck by lightning using various experimental and numerical simulations^37–39^. One study^40^ determined the effects of lightning discharge on polymer composite materials routinely used in the construction of wind turbines and showed that the thermal damage imposed on these materials was a function of non-uniform heat flux, which itself is determined by the lightning current and the discharge channel radius. We adapt the calculations used in these previous studies of lightning-induced damage to polymer composites to determine effects on materials of simplified composition and structure. A total of eight current impulse experiments were performed. Pseudo-ash samples (not natural volcanic ash) were used to represent simplified ash compositions and grain size distributions. Two different samples composed of 1 μm powders of SiO_2_, and Fe_2_O_3_ were utilised, with melting points of 1710 °C and 1560 °C, respectively. Natural volcanic ash will include mineral and glass phases containing these compounds, in addition to many others not specifically examined here (e.g., CaO, Na_2_O_3_, K_2_O). The two powders used allow examination of lightning discharge on controlled material compositions with distinguishable melting temperatures. SiO_2_ is the primary component of silicate magmas and Fe_2_O_3_ was used because there may be an observable colour change between the more oxidised form, which is red, and a reduced form (FeO or Fe), which can be black in colour, potentially enabling identification of chemical reduction following lightning discharge.

## Results

### Waveform calculations

We use modified forms of equations established by previous studies in the analysis of lightning discharge effects on various materials utilised in construction of aircraft skins and wind turbines. However, in those studies, the impulse generator typically acts as the cathode and the material subjected to the current impulse acts as the anode. Consequently, thermo-mechanical effects are imposed on the anode (e.g., carbon fiber/epoxy laminates). In the study presented here, the pseudo-ash samples are placed on an aluminum (Al) alloy plate atop the cathode, which acts as the source of the current impulse, and thus, the arc travels through the pseudo-ash before reaching the attachment point.

Using equations described in previous models^30^, the arc channel radius (*R*) is calculated:

1 $$R=.097({{I}_{max}}^{1/3}){t}^{1/2}$$

Where *R* is the channel radius in meters, *I*_*max*_ is the peak current in Amperes, and *t* is time in seconds. The waveforms recorded by the oscilloscope during the experiments were utilised to determine the current value (*I*) as a function of experiment duration, and thus, *I* varies with *t*. The maximum heat flux (*Q*_*max*_) in W/m^2^ within the arc channel is then calculated from the current (at each point in time) and the channel radius. Although the thermal flux on both the cathode and anode are of the same order of magnitude, the cathode will experience a slightly higher *Q*_*max*_, which is approximated by the Richardson – Dushman formula^38^:

2 $${Q}_{max}=\frac{24I}{\pi {R}^{2}}$$

Using *Q*_*max*_, the heat flux (*Q*) can be determined as a function of distance (*r*), in meters, from the axis of the arc channel if *r* is less than *R*:

3 $$Q={Q}_{max}{e}^{(\mathrm{ln}(0.1)/{R}^{2}){r}^{2}}$$

Equation 3 allows calculation of the thermal flux in the radial dimension surrounding the arc channel axis. Studies simulating lightning discharge on composite materials will typically utilise a numerical solution with a moving thermal boundary to model the thermo-mechanical damage on subjected samples in three dimensions, as the composites of interest are layered materials^37–40^. For the purposes of this study, which seeks to constrain effects on the pseudo-ash lying between the cathode and anode, we use a very simplified form of Fourier’s Law, under the assumption that all pseudo-ash lies within the arc channel (based on experimental setup) and heat will be transferred through the thin Al plate in one direction. Typically, the discharge would be treated as a uniform cylinder surrounding the channel axis, and the temperature variations would be calculated in a cylindrical coordinate system^15^. Instead, we are interested in the instantaneous temperature change in a two-dimensional circle surrounding the pseudo-ash. Thus, the change in temperature (Δ*T*) through the Al plate is determined by:

4 $${\rm{\Delta }}T=\frac{Q}{k}z$$

where *k* is the thermal conductivity of the Al alloy (170 W/m^2^) and *z* is the average thickness of the Al plate (0.75 mm). Values obtained using eq. 4 were compared with measurements conducted on the samples using scanning electron microscope (SEM) images and photographs, both of which were processed with ImageJ freeware (https://imagej.nih.gov/ij/). The pseudo-ash grains will, of course, have their own thermal conductivity and temperatures may vary from grain to grain depending on their proximity to each other, extent of aggregation, and contact with the Al plate. This complexity drives the motivation for calculating the thermal flux through the Al plate, rather than through the pseudo-ash grains themselves.

### Effects of current impulse

**Figure 2:**
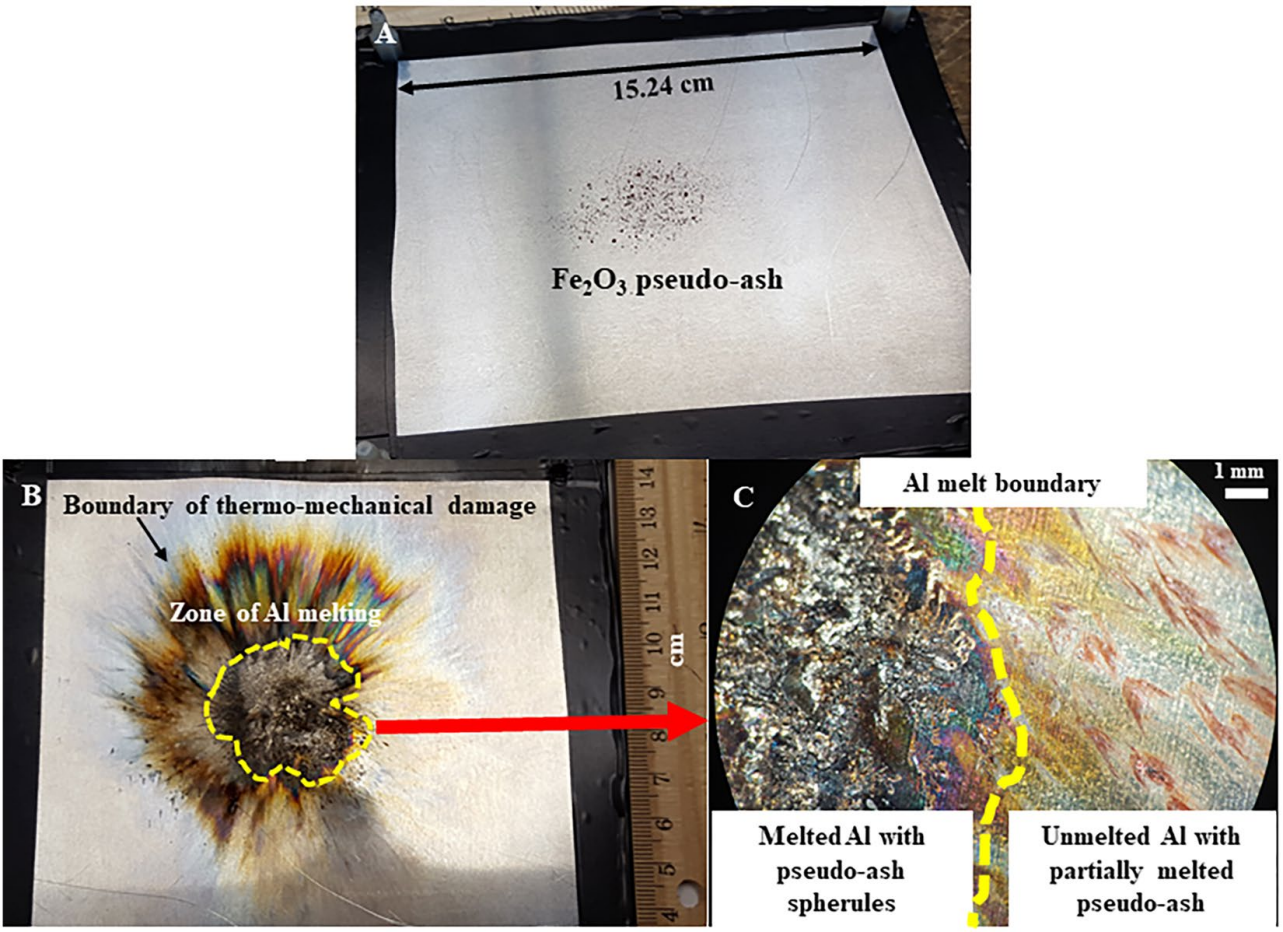
Photographs of experimental samples. (**A**) Pre-experimental photograph of Al plate dusted with Fe_2_O_3_ pseudo-ash; (**B**) Post-experimental photograph of same sample, showing the arc discharge point surrounded by a zone of melted Al outlined in yellow, with the estimated boundary of the entire arc channel indicated by a zone of discolouration; and (**C**) higher magnification view of boundary between melted Al and unmelted Al covered with Fe_2_O_3_ pseudo-ash expelled radially from the arc channel by the rapid expansion of heated air.

**Figure 3:**
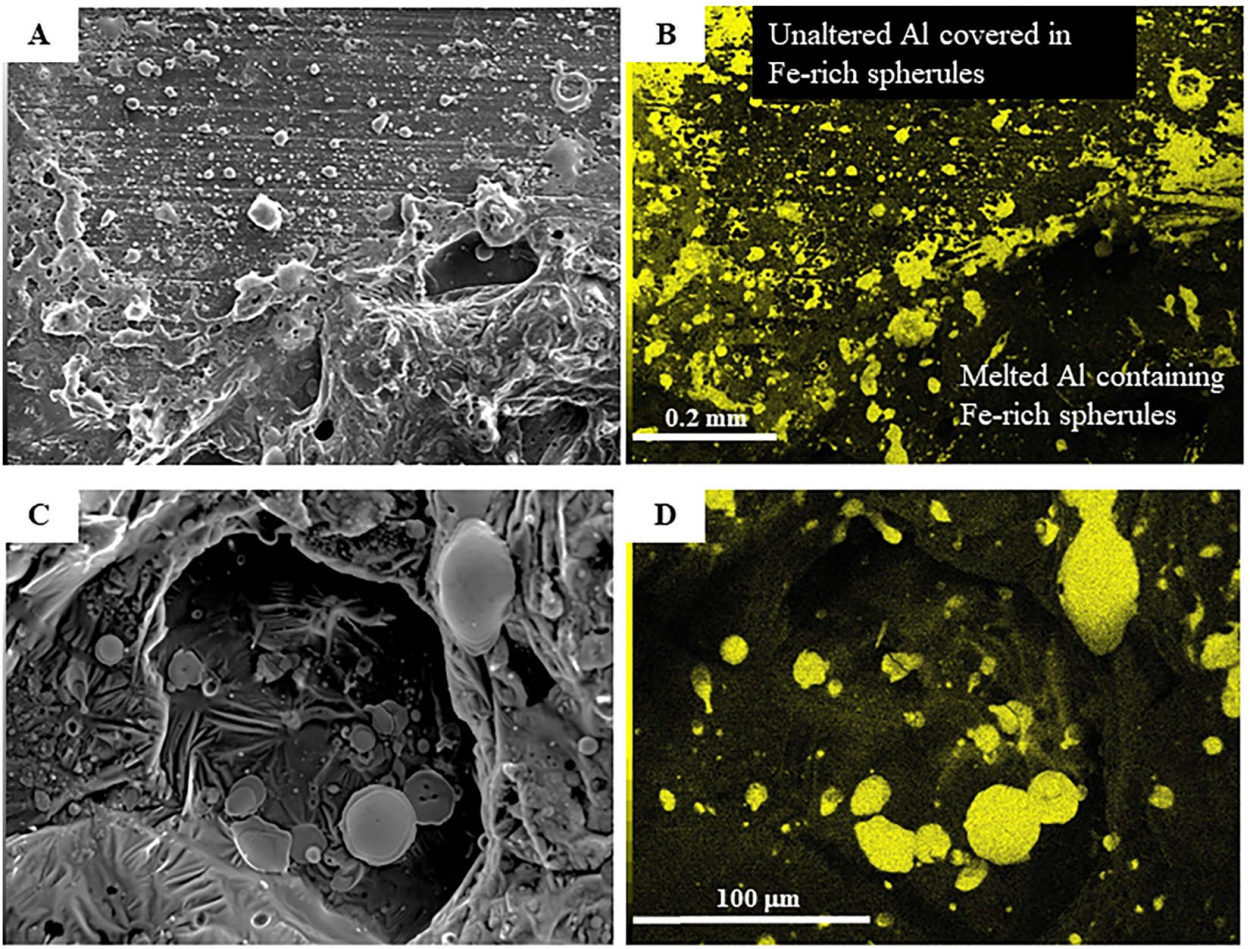
Scanning electron microscope images of post-experimental samples. (**A**) Secondary electron image of boundary between the melted Al plate in the lower part of the image and the unmelted portion in the upper part of the image, which is covered in scattered spherules and amorphous melted pieces of Fe-rich phases; (**B**) Fe X-Ray map of same area; (**C**) Higher magnification view of melted Al zone, containing spherules and flattened discs of pseudo-ash; (**D**) Fe X-Ray map of same area. Scales are the same in image (**A**) and (**B**), and also in image (**C**) and (**D**).

Images of the Al plates and pseudo-ash exposed to high-current impulses (referred to as *shots*) are presented in Fig. 2. At the discharge point, the Al plate is noticeably melted and deformed (Fig. 2B and C), in some cases forming spherules. Within this melted Al, pseudo-ash is present. For shot 8, portions of the Fe_2_O_3_ powder have been formed into smooth spherules ranging from 1 μm to 34 μm in diameter within 15 mm of the arc channel axis (Fig. 3). These spherule sizes are similar to those measured in both high-voltage flashover experiments and in natural ash fall deposits following volcanic lightning discharge^1^. For 20 measured spherules, the average diameter is 12 μm, but this average does not include the smallest spherules formed from melting and re-solidification of a single particle (~1 μm). The measured spherules result from the melting of aggregated particles. SEM images of the pseudo-ash obtained prior to the experiments show that single particles had a range of shapes and were often aggregated (Supplementary Information, Fig. S1), yet the surfaces of these individual particles and aggregates are not smooth like the post-experimental spherules. Evidence of reduction was ambiguous, as Fe-rich spherules within the Al were dark red to blue/black in colour under optical microscopy, but limited sample size inhibited chemical analysis to directly confirm or refute reduction of the Fe_2_O_3_ into other Fe phases. Other morphologies formed from melting and re-solidification of the pseudo-ash include teardrops and ‘doughnuts’ (Fig. 3), the former of which is typical in volcanic eruptions of low viscosity magmas (Pele’s tears)^41^ and the latter of which is common during arc welding (*Chris Bailey*, *Nucor Steel Tuscaloosa*, *personal comm*.*)*. In some cases, the pseudo-ash was launched from the surface of the Al plate and landed in a different location while still molten, as evidenced by flattened discs of Fe-rich particles (Fig. 3C and D). Similar textures were observed in high-voltage flashover experiments of insulators coated with volcanic ash^1^. Spherules were not observed following analysis of the shot 5 sample (SiO_2_), because the discharge point occurred close to the edge of the Al plate and the pseudo-ash was located near the plate center. However, all shots resulted in thermo-mechanical damage to the Al plate over a similar area.

In addition to forming spherules due to the high temperatures generated, the pseudo-ash is also displaced from its initial location. The rapid heating of the ambient air during the current impulse causes expansion^37–39^, which in nature contributes to thunder following lightning discharge in thunderstorms^13^. In the experiments presented here, this rapid expansion expels most of the pseudo-ash from the axis of the arc channel. This is indicated by the presence of both pseudo-ash smeared out radially from the discharge point and spherules deposited beyond the zone of thermo-mechanically altered Al (Fig. 2C). SEM X-ray maps indicate that the pseudo-ash was partially melted during this process, as the surfaces of the aggregates are smooth and single particles cannot be differentiated (Fig. 3). Because the arc channel was not perfectly symmetric, the expelled pseudo-ash was not uniformly distributed around the boundary of the arc channel (Fig. 2B). These results suggest that high-current discharge will morphologically alter the exposed ash and simultaneously expel it radially from its original location within the arc channel while the ash is still at a temperature above its melting point.

**Figure 4:**
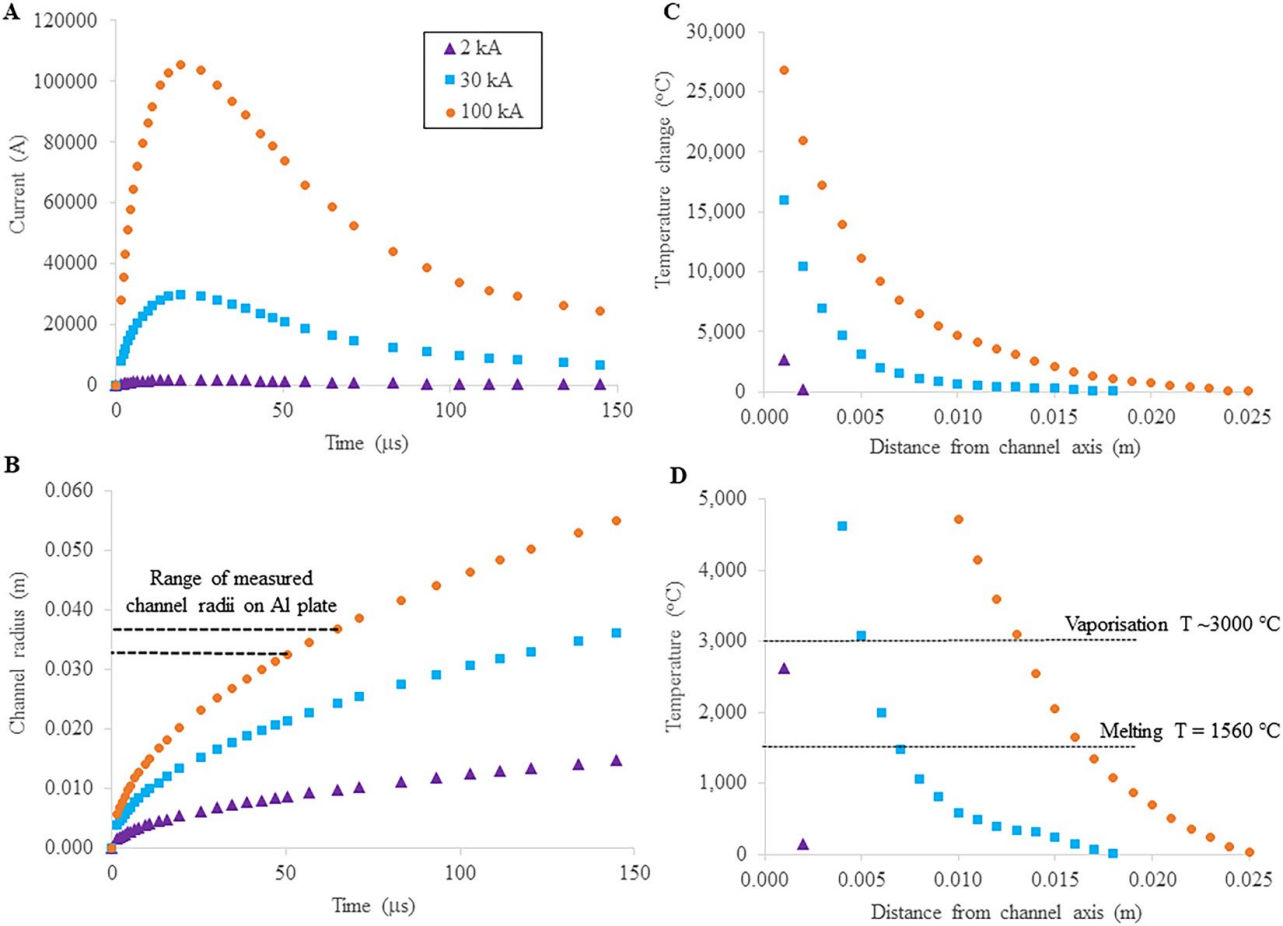
Results of current waveform analyses. (**A**) Current waveform for one (shot 8, using Fe_2_O_3_ pseudo-ash) of the eight high-current impulse experiments performed in this study. Experiments were performed using a peak current of ~100 kA, and calculations are also provided for 30 kA and 2 kA for comparison although these peak currents were not directly tested; (**B**) Calculated arc channel radius as a function of experimental duration (error <1.6%) with dashed horizontal lines bounding the range of channel radii as measured on the Al plates for the ~100 kA current impulses; (**C**) Calculated temperature change with distance from the axis of the arc channel; and (**D**) temperature of the Al plate, relative to an ambient temperature of 25 °C, with distance from the axis of the arc channel. The data displayed in (**D**) are for temperatures relevant to minerals and glasses common in volcanic ash. The two horizontal, dashed lines indicate the melting temperature of 1560 °C and vaporisation temperature of ~3000 °C, respectively, for the pseudo-ash. Consequently, the ideal melting zone occurs over a distance of only 3 mm for a ~100 kA current impulse, ~2 mm for a 30 kA current impulse, and ~1 mm for a 2 kA current impulse. Errors for all values are within the size of the symbols. Results of all six recorded shots conducted with a ~100 kA current impulse and their associated errors can be found in the Supplementary Information, Table S1.

Five shots were utilised for the waveform calculations; shots 5 and 8 in addition to three conducted on the bare Al plates. For the purpose of analysis, shot 8 is the focus, because this was a sample of Fe_2_O_3_, lightly dusted on the Al plate, allowing straightforward examination of changes to particle morphology. Additionally, two of the eight shots were not recorded by the oscilloscope. The current waveform for shot 8 is shown in Fig. 4A as a function of time in μs. Using eq. 3, the heat flux was calculated for an expanding channel radius (Fig. 4B) and the temperature change as a function of distance from the axis of the channel was determined with eq. 4 (Fig. 4C). For shots 5 and 8, assuming an ambient temperature of 25 °C, eq. 4 provides temperature values that exceed the melting point of SiO_2_ and Fe_2_O_3_ at a distance of 14 mm and 16 mm from the channel axis, respectively (Fig. 4D). Temperatures exceed the melting point of the Al alloy (640 °C) at a distance of 17 mm and 20 mm, respectively. These values agree with average Feret radii of melted zones within the Al plate, ranging from 13.0 mm to 17.4 mm with an average of 15.0 (±2) mm for all measurements (n = 25). The total radius of the Al plate observed to undergo thermo-mechanical damage was measured to be 33.0 mm and 37.3 mm for shots 5 and 8, respectively, based on areas of Al discolouration, deformation, and melting (Fig. 2B) and these areas were interpreted as the arc channel radius, as there was no evidence of thermo-mechanical damage to the Al plate outside of these radial distances. This was confirmed by calculations, which resulted in temperatures of 450 °C at *R* = 33 mm and 230 °C at *R* = 37 mm for shots 5 and 8, respectively, which are both below the melting temperature of Al at these distances from the channel axis. Measured radii of the interpreted arc channel correlate well with estimates of CG return stroke channel radii of ~3 cm^26,42^, although other studies have suggested radii as small as 0.5 cm^27^. Calculated results for the other waveforms are similar (Supplementary Information, Table S1).

### Temperature changes

Waveform calculations for all six of the recorded high-current impulse experiments reveal that near the axis of the arc channel, temperatures are well above those required to melt most natural minerals and glasses found within volcanic ash. Temperature rapidly drops off with distance from the channel axis, falling to <2000 °C at a radius of >15 mm. Similar results from other lightning simulation studies^40^, show a rapid decrease in temperature over a small radial distance (~1 cm). Natural lightning temperatures are estimated to range up to 30000 °C, and temperatures achieved in the six current impulse experiments were calculated to be from 17200 °C up to 26800 °C at a distance of 1 mm from the channel axis. Depending upon the composition of ash grains exposed to lightning discharge, some materials may reach melting temperatures while others do not, even when located within the same region of the discharge channel, but near the channel axis all materials will be similarly affected due to temperatures in excess of 20000 °C. For the purposes of these experiments, we employ a current impulse with a peak value of ~100 kA. As described previously, the median value of CG return strokes from thunderstorms will reach an *I*_*max*_ of less than half this, and for volcanic lightning, over an order of magnitude less. We utilise the ~100 kA *I*_*max*_, to represent the *upper limit* during volcanic lightning discharge, based on the values for thunderstorm CG return strokes and the limited dataset regarding the *I*_*max*_ of volcanic plume lightning (which may reach values closer to thunderstorm lightning due to greater lengths and timescales compared to near-vent lightning). For comparison, we performed the same calculations, changing only the *I*_*max*_ to values matching previous studies (30 kA and 2 kA), and keeping all other parameters consistent (Fig. 4). For an *I*_*max*_ of ~100 kA, waveform calculations indicate temperatures are high enough to melt igneous minerals and glasses between 15 and 16 mm from the axis of the channel, and rapidly fall off beyond this distance. However, at only 12 to 13 mm from the channel axis, temperatures exceed those required to vaporise the same phases (~3000 °C)^43,44^. The difference between melting the ash and vaporising it occurs over a length scale of only 3 mm in these current impulse experiments. For an *I*_*max*_ of 30 kA, the zone of melting is ~2 mm and for an *I*_*max*_ of 2 kA, the zone of melting is ~1 mm (Fig. 4D). A discharge with an *I*_*max*_ of 2 kA may vaporise ash at the channel center, and an *I*_*max*_ of 30 kA can certainly reach the necessary temperature (Fig. 4D). If *I*_*max*_ values are similar to median values of CG return strokes^32^, ash may be either melted or vaporised (or dissociated) depending on position within the channel. However, for *I*_*max*_ values determined thus far for lightning discharge^28^, volcanic ash may be melted, and/or vaporised, if within 1 mm of the channel axis. Of course, volcanic ash samples will be composed of various minerals and/or elemental oxides that will have different melting and vaporisation temperatures than the samples used here. Generally, as melting temperature decreases, so does vaporisation temperature, so the range between these temperatures should remain relatively consistent. Thus, regardless of the *I*_*max*_ and grain composition, there is a limited zone within the discharge channel that reaches temperatures sufficient to melt ash particles.

## Discussion

The approximate volume of a discharge channel with a core radius of 30 mm and a length of 100 m would be 0.28 m^3^, and ash located within only 10% of this volume would be melted, but not vaporised. However, for an *I*_*max*_ of only 2 kA, using a smaller core channel radius of 10 mm, the discharge channel volume would be 0.031 m^3^ and 10% or less of this volume would reach temperatures sufficient to melt ash. Consequently, for the ash particle concentrations of eruptive columns previously given, only tens to a few tens of thousands of those particles would potentially “survive” the discharge event. Those ash particles within the ideal melting zone of the discharge channel (one-tenth or less of the radius) would simultaneously be expelled by the rapid expansion of heated air and eventually deposited amongst other unmodified ash grains.

To estimate the potential amount of ash transformation during an explosive volcanic eruption, we use the April 22–23, 2015 eruption of Calbuco volcano in Chile as an example. This eruption consisted of two explosive phases, during which over 1100 lightning flashes were detected^45^. Using the same discharge channel volume as above (0.28 m^3^), this results in a total lightning channel volume of roughly 308 m^3^ throughout the course of the eruption. Based on the location of detected lightning flashes (~15 km radial distance from the vent)^45^ and direct observations of the column height (15 km)^45,46^, the total volume of the eruption column is estimated at 3.53 × 10^12^ m^3^. Thus, throughout the eruption, the proportion of the total lightning channel volume at a temperature conducive to forming LIVS, assuming an *I*_*max*_ of ~100 kA, represents only 8.7 × 10^−10^% of the entire eruptive column. If we utilise calculations using an *I*_*max*_ of 2 kA, this value decreases by roughly an order of magnitude to 9.7 × 10^−11^%. Lightning channel volumes may be smaller or larger than the values used here, particularly for plume lightning that occurs farther from the vent (which tends to form longer arcs) or for relatively smaller, near-vent discharges. Additionally, grain size analyses of the Calbuco deposits indicate that only 10–15% of the erupted material in medial to distal locations from the vent was a size (<150 μm; 2.74 ϕ)^46^ conducive to melting over the timescales of lightning discharge^36^. Consequently, the calculations presented here represent merely a first order assessment of the potential proportion of volcanic ash that may retain evidence of being subjected to lightning discharge.

Simulating both the high currents and high voltages of natural lightning cannot be achieved simultaneously in a laboratory setting, but the methods employed in this study are consistent with those outlined by the International Electrotechnical Commission regarding characterisation of the damage to an object (e.g., aircraft material, wind turbine) struck by lightning^47,48^. Both channel radius and temperature will decrease with lower *I*_*max*_ values (Fig. 4), and total timescales for lower *I*_*max*_ discharges will also be reduced. The calculations in this study use a resolution of 1 mm per data point to determine variations in heat flux and temperature as a function of distance from the channel axis. Current impulses were not directly performed at 30 kA and 2 kA, but future experiments will utilise a lower *I*_*max*_ and an improved resolution for the length scale to refine the models.

The rapid air expansion that results from the discharge will expel the molten ash from the channel, allowing it to cool during atmospheric transport, resulting in the formation of LIVS observed in ash fall deposits. Because these experiments could not simulate a continuing current for tens of milliseconds, volcanic ash may undergo longer durations of heating during lightning discharge, regardless of peak current. This is supported by spectral studies of natural CG return strokes, which show that temperatures will decrease at a lower rate than the current^33^. Video analysis of volcanic lightning at Sakurajima reveals timescales of roughly 0.1 to 8 ms (average of 2.5 ms) for the lightning discharge and following afterglow^25,28,36^. The afterglow is interpreted to result from the relatively slower cooling (compared to heating) of ash particles subjected to lightning in the eruptive column^36^. For the upper limit of the discharge timescales observed at Sakurajima, modeling results indicate that ash particles <150 μm can sufficiently melt, deform under surface tension, and re-solidify into LIVS as they cool^36^.

Both natural lightning (including volcanic lightning) and high-current impulse experiments are dynamic physical events, and as many previous studies have indicated, it is challenging to constrain all the variables involved. Calculations presented here use a simplified form of Fourier’s Law and a constant value for the thermal conductivity of the Al plate, assuming heat will be transferred directly to the pseudo-ash. However, thermal conductivity will vary as a function of temperature and this study does not incorporate any heat transferred from the surrounding air to the pseudo-ash, but this will be negligible during the timescale of the current impulse. The thermal and electrical conductivity of the air gap between the cathode (Al alloy plate) and anode (steel plate) within the experimental apparatus will both vary due to potential vaporisation of the Al and pseudo-ash. Additionally, use of the conductive Al plate may affect the size of the resulting arc channel. Thermal conductivity of air is several orders of magnitude lower than Al metal, and volcanic ash within explosive eruption plumes are suspended in a medium of ambient air mixed with other ash particles (of different compositions and grain sizes) and volatiles (e.g., H_2_O, SO_2_) released during the explosion, all at various temperatures. This mixture of materials will result in complex and spatially variable thermal conductivity during volcanic lightning discharge, causing an equally complex change in temperature throughout the discharge channel, constraint of which is beyond the scope of this study. The potential vaporisation of ash during lightning discharge may further impact the electrochemical environment of the local atmosphere, which is a topic for future studies and may pose important implications for changes in atmospheric chemistry as the result of lightning discharge in thunderstorms containing mineral dusts.

The results presented here are not only relevant for an improved understanding of volcanic lightning and evidence of this phenomenon in ash fall deposits, but also for atmospheric science and material response to lightning strikes. The experimental high-current impulses clearly damaged the Al plates on which the pseudo-ash samples were resting (Fig. 2). The transient electromagnetic field of a lightning discharge, also called an electromagnetic pulse, can induce a secondary current in an electrically conductive material that is struck. The interaction of the electromagnetic fields generated by the primary current (lightning), and the induced current, creates a repulsive force that leads to deformation of these materials^49^, while heat resulting from the plasma also causes thermal damage. The combined effects result in the area of thermo-mechanical damage on the Al plates used in the experiments presented here (Fig. 2B and C). The same effects can occur to electrical infrastructure struck by lightning, as the induced current and resulting deformation can lead to the destruction of electronics. We interpret the melted and discoloured areas of the Al plate as the total arc channel for purposes of comparing post-experimental sample measurements to calculations. However, it should be noted that portions of the discoloured plate may result from deposition of the vaporised materials after they have combined with ambient oxygen. The final depositional location of vaporised phases following both high-current impulse experiments and volcanic lightning is a topic of continuing research.

Using high-current impulse experiments, this study provides the distance from the axis of the lightning discharge channel at which volcanic ash will be morphologically transformed into LIVS. Within the channel, there is an ideal melting zone that represents roughly 10% or less of the total channel radius in which the ash particles will be melted, regardless of the peak current value. Additionally, the rapid air expansion generated by the discharge expels ash from the channel while still molten, permitting it to cool during transport in the ambient atmosphere before deposition. A limited zone of melting within, coupled with expulsion from, the discharge channel explains why ash fall deposits typically contain few LIVS within a population of unaltered ash particles, even though volcanic lightning is a common occurrence during explosive eruptions. These results pose important implications for the effect of lightning on atmospheric properties, infrastructure, and natural/manufactured materials.

## Materials and Methods

The centers of 232 cm^2^, 0.75 mm-thick 3105-H24 aluminum (Al) alloy plates, with a melting temperature of 640 °C, were lightly dusted with pseudo-ash. The plates are dominantly composed of Al (98%) with trace amounts of other metals including Mn, Mg, Cu, and Zn. The alloy plates were used due to their high thermal/electrical conductivity, low melting point relative to the pseudo-ash, and ability to structurally support the ash samples within the experimental apparatus. Pseudo-ash samples were deliberately placed in the centers of these plates under the assumption that the current impulse would be generated from this area, and all the pseudo-ash would be surrounded by the arc channel.

### Experimental Design

Lightning discharge simulation experiments were conducted at the High Voltage Laboratory at Mississippi State University. The Marx bank current impulse generator was designed at Mississippi State and configured to assess lightning-induced damage on different test articles. The generator consists of six high energy density Aerovox capacitors with a capacitance of 50 μF and a charging voltage of 44 kV, so that each can store up to 50 kJ of energy (Supplementary Information, Fig. S2A). When the charging of the capacitors is complete, the capacitor bank is switched with a triggered Maxwell 40200 rail gap switch that imposes the open-circuit voltage of the charged capacitors to the air gap formed between the semispherical output electrode and the surface of the test article. The Al alloy plates dusted with pseudo-ash were bolted to the underside of a 76 cm by 76 cm steel plate, and placed in direct contact with the output electrode (Supplementary Information, Fig. S2B). Thus, the high current discharge originates from the output electrode, travels through the Al plate and the pseudo-ash coating the surface, and arcs across a 2.5 cm spark gap to the steel plate above. Copper braids then return the current through the structure of the impulse generator back to the capacitors. Current waveforms are recorded on a Tektronix TDS7104 1 GHz bandwidth digital phosphor oscilloscope at a sampling interval of 2 ns. A total of eight current impulses were performed; three on bare Al plates and five using plates dusted with pseudo-ash. Samples were photographed prior to and following experiments. The Al plates subjected to the discharge were then sectioned to permit examination with the JEOL JSM 6010Plus/LA scanning electron microscope (SEM) housed in the Tephra Laboratory located within the Department of Geological Sciences at the University of Alabama. Secondary electron images and element X-ray maps were acquired at an accelerating voltage of 20 kV to examine morphological alteration of both the Al plate and the pseudo-ash. Evidence of lightning-induced spherules created from the pseudo-ash particles were identified and their diameters were measured using Image-J freeware (https://imagej.nih.gov/). Additionally, variations to particle and plate morphology were determined with distance from the arc channel axis and compared to results derived from the waveform calculations.

## Electronic supplementary material

Supplementary Information
