# Supplementary material for: The Elusive Evidence of Volcanic Lightning
Source: Sci Rep. 2017 Nov 14;7:15508. doi: 10.1038/s41598-017-15643-8 (PMC5686202; doi:10.1038/s41598-017-15643-8)
Supplement: Supplementary file 1 — Supplementary Information [file 41598_2017_15643_MOESM1_ESM.pdf]

The Elusive Evidence of Volcanic Lightning

K. Genareau[1*], P. Gharghabi[2], J. Gafford[3], and M. Mazzola[2]

[1]Department of Geological Sciences, University of Alabama, Box 870338, Tuscaloosa, Alabama 35487, USA

[2]Department of Electrical and Computer Engineering, Mississippi State University, Starkville, Mississippi 39762 USA.

[3]Center for Advanced Vehicular Systems at Mississippi State University, Starkville, Mississippi 39759 USA.

*Corresponding author: Kimberly Genareau (kdg@ua.edu)

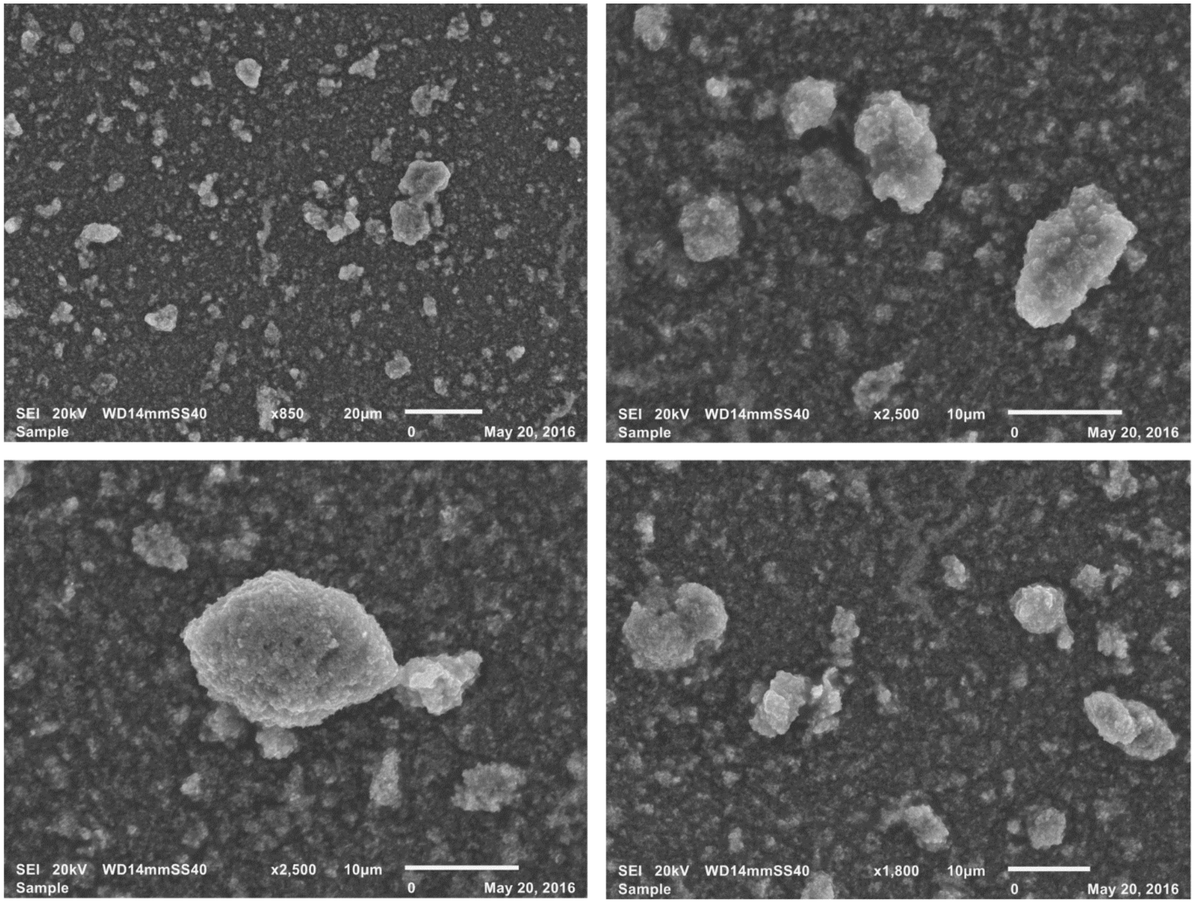

Figure S1. Secondary electron images of pre-experimental Fe_2O_3 pseudo-ash. Images show single 1 μm particles and 10-20 μm aggregates of particles with rough surfaces. Post-experimental samples reveal smoothed aggregate surfaces as a result of melting in the current impulse discharge channel.

Table S1. Waveform calculation results for all shots. See text of manuscript for utilized equations.

Negative temperature values correspond to radii at which the Al plate returned to ambient temperature (25 ^{o}C).

For R, Q_{max}, and Q, the uncertainty provided is the maximum and progressively decreases with lower values of the variable.

Shot 2

	r (m)	*I_{max} (Amps)*	*I (Amps)*	*time (s)*	*R (m)*	*Q_{max} (W/m^2)*	*Q (W/m^2)*	*T (°C)*	*error in T (°C)*
Uncertainty		*± 100 Amps*	*± 100 Amps*	*± 0.5E-06 s*	*<1.6%*	*<0.03%*	*<0.04%*		
	0.001	102400	0	0	0	N/A	N/A	N/A	N/A
	0.002		30316	1.6E-06	0.006	7.2E+09	5.4E+09	23941	773
	0.003		41768	2.5E-06	0.007	6.3E+09	4.2E+09	18438	596
	0.004		55242	4.2E-06	0.009	4.9E+09	3.2E+09	13957	451
	0.005		61979	5.3E-06	0.010	4.3E+09	2.5E+09	11101	359
	0.006		69389	6.5E-06	0.012	4.0E+09	2.1E+09	9234	298
	0.007		80168	8.5E-06	0.013	3.5E+09	1.8E+09	7910	256
	0.008		84884	9.6E-06	0.014	3.3E+09	1.6E+09	6652	215
	0.009		90274	1.1E-05	0.015	3.0E+09	1.3E+09	5670	183
	0.010		97684	1.5E-05	0.018	2.4E+09	1.1E+09	4827	156
	0.011		101726	1.9E-05	0.020	1.9E+09	9.7E+08	4027	130
	0.012		97684	2.8E-05	0.024	1.3E+09	7.3E+08	2967	96
	0.013		92968	3.3E-05	0.026	1.1E+09	5.9E+08	2354	76
	0.014		88253	3.6E-05	0.027	9.0E+08	4.9E+08	1905	62
	0.015		80168	4.3E-05	0.030	7.0E+08	3.9E+08	1429	46
	0.016		74105	4.7E-05	0.031	5.8E+08	3.2E+08	1122	36
	0.017		67368	5.3E-05	0.033	4.7E+08	2.6E+08	850	27
	0.018		60632	5.9E-05	0.035	3.8E+08	2.1E+08	622	20
	0.019		53221	6.7E-05	0.037	3.0E+08	1.6E+08	425	14
	0.020		44463	7.8E-05	0.040	2.1E+08	1.2E+08	236	8
	0.021		40084	8.4E-05	0.042	1.8E+08	9.8E+07	141	5
	0.022		37053	9.0E-05	0.043	1.5E+08	8.4E+07	76	2
	0.023		33179	9.9E-05	0.045	1.2E+08	6.8E+07	7	0
	0.024		29305	1.1E-04	0.048	9.9E+07	5.5E+07	-52	2
	0.025		26947	1.2E-04	0.049	8.5E+07	4.7E+07	-89	3
	0.026		24253	1.3E-04	0.052	6.9E+07	3.9E+07	-125	4
	0.027		22232	1.4E-04	0.054	5.8E+07	3.3E+07	-152	5
	0.028		20884	1.6E-04	0.057	5.0E+07	2.8E+07	-172	6
	0.029		19032	1.8E-04	0.061	3.9E+07	2.3E+07	-195	6
	0.030		18358	2.1E-04	0.065	3.3E+07	2.0E+07	-208	7
	0.031		18189	2.3E-04	0.068	3.0E+07	1.9E+07	-215	7

Shot 3									
	***r* (m)**	***I*** ***I_{max}* (Amps)**	***I* (Amps)**	***time* (s)**	***R* (m)**	***Q_{max}* (W/m^2)**	***Q* (W/m^2)**	***T* (°C)**	***error in T* (°C)**
Uncertainty		***± 100 Amps***	***± 100 Amps***	***± 0.5E-06 s***	***<2.0%***	***<0.03%***	***<0.04%***		
	0.001	101600	0	0	0	N/A	N/A	N/A	N/A
	0.002		24223	1.3E-06	0.005	7.1E+09	5.0E+09	22036	712
	0.003		32297	2.2E-06	0.007	5.6E+09	3.5E+09	15294	494
	0.004		42389	3.1E-06	0.008	5.1E+09	2.9E+09	12516	404
	0.005		51136	4.3E-06	0.009	4.5E+09	2.3E+09	10056	325
	0.006		59211	5.4E-06	0.011	4.1E+09	1.9E+09	8330	269
	0.007		68630	6.6E-06	0.012	3.9E+09	1.7E+09	7224	233
	0.008		76705	8.4E-06	0.013	3.4E+09	1.4E+09	6171	199
	0.009		85452	1.0E-05	0.015	3.1E+09	1.3E+09	5410	175
	0.010		92853	1.3E-05	0.016	2.7E+09	1.1E+09	4718	152
	0.011		97563	1.6E-05	0.018	2.3E+09	9.7E+08	4053	131
	0.012		100927	2.0E-05	0.020	1.8E+09	8.3E+08	3433	111
	0.013		98909	2.6E-05	0.023	1.4E+09	6.8E+08	2746	89
	0.014		93526	3.2E-05	0.026	1.1E+09	5.5E+08	2143	69
	0.015		86797	3.8E-05	0.028	8.5E+08	4.4E+08	1661	54
	0.016		78050	4.5E-05	0.030	6.5E+08	3.4E+08	1228	40
	0.017		70649	5.1E-05	0.032	5.2E+08	2.7E+08	922	30
	0.018		64593	5.6E-05	0.034	4.3E+08	2.2E+08	706	23
	0.019		58538	6.2E-05	0.036	3.5E+08	1.8E+08	519	17
	0.020		50464	7.1E-05	0.038	2.6E+08	1.4E+08	331	11
	0.021		46426	7.6E-05	0.040	2.3E+08	1.2E+08	232	7
	0.022		41717	8.4E-05	0.041	1.9E+08	9.7E+07	136	4
	0.023		35829	9.5E-05	0.044	1.4E+08	7.5E+07	38	1
	0.024		30278	1.1E-04	0.047	1.0E+08	5.7E+07	-42	1
	0.025		26914	1.2E-04	0.050	8.3E+07	4.6E+07	-91	3
	0.026		24223	1.4E-04	0.053	6.6E+07	3.8E+07	-128	4
	0.027		21195	1.6E-04	0.057	5.0E+07	3.0E+07	-164	5
	0.028		20185	1.7E-04	0.059	4.4E+07	2.6E+07	-180	6
	0.029		19513	1.9E-04	0.062	3.9E+07	2.4E+07	-193	6
	0.030		18503	2.1E-04	0.065	3.3E+07	2.0E+07	-207	7
	0.031		18335	2.3E-04	0.068	3.0E+07	1.9E+07	-215	7

Shot 4									
	***r* (m)**	***I*$_{max}$ (Amps)**	***I* (Amps)**	**time (s)**	***R* (m)**	***Q*$_{max}$ (W/m^2)**	***Q* (W/m^2)**	***T* (°C)**	**error in *T* (°C)**
Uncertainty		**± 100 Amps**	**± 100 Amps**	**± 0.5E-06 s**	**<1.3%**	**<0.03%**	**<0.03%**		
	0.001	104000	0	0	0	N/A	N/A	N/A	N/A
	0.002		26338	2.0E-06	0.006	4.9E+09	3.9E+09	17216	556
	0.003		37143	3.2E-06	0.008	4.2E+09	3.1E+09	13617	440
	0.004		47273	4.2E-06	0.009	4.2E+09	2.7E+09	11877	384
	0.005		55377	5.1E-06	0.010	4.0E+09	2.3E+09	10064	325
	0.006		63481	6.6E-06	0.012	3.5E+09	1.9E+09	8339	269
	0.007		72260	7.8E-06	0.013	3.4E+09	1.7E+09	7286	235
	0.008		81714	9.9E-06	0.014	3.0E+09	1.5E+09	6330	204
	0.009		86442	1.1E-05	0.015	2.9E+09	1.3E+09	5406	175
	0.010		91844	1.3E-05	0.016	2.6E+09	1.1E+09	4663	151
	0.011		99273	1.6E-05	0.018	2.2E+09	9.8E+08	4100	132
	0.012		103325	2.1E-05	0.021	1.8E+09	8.5E+08	3492	113
	0.013		101974	2.6E-05	0.023	1.4E+09	7.0E+08	2824	91
	0.014		97922	3.1E-05	0.025	1.2E+09	5.8E+08	2281	74
	0.015		89818	3.8E-05	0.028	8.7E+08	4.5E+08	1715	55
	0.016		81714	4.5E-05	0.031	6.7E+08	3.6E+08	1293	42
	0.017		74286	5.0E-05	0.032	5.5E+08	2.9E+08	988	32
	0.018		66182	5.7E-05	0.034	4.3E+08	2.3E+08	718	23
	0.019		58078	6.5E-05	0.037	3.3E+08	1.8E+08	496	16
	0.020		49299	7.4E-05	0.039	2.4E+08	1.3E+08	301	10
	0.021		41364	8.6E-05	0.042	1.8E+08	1.0E+08	150	5
	0.022		35792	9.7E-05	0.045	1.4E+08	7.8E+07	51	2
	0.023		31065	1.1E-04	0.047	1.1E+08	6.1E+07	-24	1
	0.024		27013	1.2E-04	0.051	8.0E+07	4.8E+07	-84	3
	0.025		23636	1.4E-04	0.054	6.2E+07	3.8E+07	-130	4
	0.026		21610	1.6E-04	0.057	5.1E+07	3.1E+07	-158	5
	0.027		19753	1.8E-04	0.061	4.1E+07	2.6E+07	-182	6
	0.028		19078	2.0E-04	0.064	3.6E+07	2.3E+07	-196	6
	0.029		18571	2.1E-04	0.066	3.2E+07	2.1E+07	-205	7
	0.030		18234	2.3E-04	0.069	3.0E+07	1.9E+07	-213	7
	0.031		17896	2.5E-04	0.072	2.7E+07	1.7E+07	-221	7

Shot 5									
	***r* (m)**	***I*$_{max}$ (Amps)**	***I* (Amps)**	***time* (s)**	***R* (m)**	***Q*$_{max}$ (W/m^2)**	***Q* (W/m^2)**	***T* (°C)**	***error in T* (°C)**
Uncertainty		***± 100 Amps***	***± 100 Amps***	***± 0.5E-06 s***	***<1.2%***	***<0.03%***	***<0.03%***		
	0.001	103200	0	0	0	N/A	N/A	N/A	N/A
	0.002		23844	2.1E-06	0.007	4.1E+09	3.9E+09	17210	556
	0.003		34625	3.2E-06	0.008	4.0E+09	3.5E+09	15202	491
	0.004		43382	4.3E-06	0.009	3.7E+09	3.0E+09	12901	417
	0.005		52819	4.8E-06	0.010	4.1E+09	2.8E+09	12215	395
	0.006		60227	5.9E-06	0.011	3.8E+09	2.4E+09	10209	330
	0.007		70327	7.6E-06	0.013	3.4E+09	2.0E+09	8743	282
	0.008		81101	9.2E-06	0.014	3.3E+09	1.8E+09	7746	250
	0.009		89164	1.2E-05	0.016	2.7E+09	1.5E+09	6478	209
	0.010		95204	1.5E-05	0.017	2.4E+09	1.3E+09	5477	177
	0.011		98552	1.7E-05	0.019	2.1E+09	1.1E+09	4669	151
	0.012		101201	2.2E-05	0.021	1.7E+09	9.3E+08	3850	124
	0.013		98447	2.7E-05	0.024	1.4E+09	7.5E+08	3041	98
	0.014		94350	3.1E-05	0.025	1.1E+09	6.1E+08	2431	79
	0.015		84826	3.9E-05	0.028	8.1E+08	4.6E+08	1761	57
	0.016		79380	4.3E-05	0.030	6.8E+08	3.8E+08	1400	45
	0.017		73260	4.8E-05	0.031	5.7E+08	3.1E+08	1095	35
	0.018		63730	5.6E-05	0.034	4.2E+08	2.4E+08	761	25
	0.019		52132	6.8E-05	0.037	2.8E+08	1.7E+08	447	14
	0.020		45981	7.5E-05	0.039	2.3E+08	1.3E+08	293	9
	0.021		41179	8.3E-05	0.041	1.8E+08	1.1E+08	182	6
	0.022		34996	9.3E-05	0.044	1.4E+08	8.2E+07	68	2
	0.023		30831	1.0E-04	0.046	1.1E+08	6.5E+07	-6	0
	0.024		24206	1.2E-04	0.051	7.2E+07	4.5E+07	-97	3
	0.025		21697	1.4E-04	0.053	5.8E+07	3.7E+07	-135	4
	0.026		19543	1.5E-04	0.056	4.8E+07	3.0E+07	-162	5
	0.027		17291	1.7E-04	0.059	3.8E+07	2.4E+07	-190	6
	0.028		16449	1.9E-04	0.062	3.3E+07	2.1E+07	-204	7
	0.029		15626	2.0E-04	0.064	2.9E+07	1.9E+07	-215	7
	0.030		15416	2.2E-04	0.067	2.6E+07	1.7E+07	-222	7
	0.031		14362	2.4E-04	0.070	2.2E+07	1.5E+07	-233	8

Shot 6									
	***r* (m)**	***I_{max}* (Amps)**	***I* (Amps)**	***time* (s)**	***R* (m)**	***Q_{max}* (W/m^2)**	***Q* (W/m^2)**	***T* (°C)**	***error in T* (°C)**
Uncertainty		***± 100 Amps***	***± 100 Amps***	***± 0.5E-06 s***	***<1.7%***	***<0.04%***	***<0.04%***		
	0.001	104000	0	0.0E+00	0.000	N/A	N/A	N/A	N/A
	0.002		25150	1.5E-06	0.006	6.0E+09	4.5E+09	19826	640
	0.003		33987	2.4E-06	0.007	5.1E+09	3.4E+09	14956	483
	0.004		42144	3.3E-06	0.008	4.7E+09	2.7E+09	11944	386
	0.005		53020	4.8E-06	0.010	4.1E+09	2.3E+09	9920	320
	0.006		60497	5.6E-06	0.011	3.9E+09	1.9E+09	8386	271
	0.007		70693	7.7E-06	0.013	3.4E+09	1.7E+09	7165	231
	0.008		80209	9.1E-06	0.014	3.2E+09	1.5E+09	6343	205
	0.009		88366	1.1E-05	0.015	2.8E+09	1.3E+09	5498	178
	0.010		95843	1.4E-05	0.017	2.5E+09	1.1E+09	4781	154
	0.011		100601	1.7E-05	0.019	2.2E+09	9.9E+08	4133	133
	0.012		103320	2.1E-05	0.021	1.8E+09	8.5E+08	3495	113
	0.013		99922	2.8E-05	0.024	1.3E+09	6.7E+08	2716	88
	0.014		93804	3.3E-05	0.026	1.0E+09	5.4E+08	2114	68
	0.015		87007	3.9E-05	0.028	8.2E+08	4.3E+08	1639	53
	0.016		79529	4.5E-05	0.031	6.5E+08	3.5E+08	1248	40
	0.017		72052	5.0E-05	0.032	5.2E+08	2.8E+08	945	31
	0.018		63895	5.7E-05	0.035	4.1E+08	2.2E+08	680	22
	0.019		55059	6.6E-05	0.037	3.0E+08	1.7E+08	448	14
	0.020		48261	7.4E-05	0.039	2.4E+08	1.3E+08	290	9
	0.021		41464	8.4E-05	0.042	1.8E+08	1.0E+08	155	5
	0.022		34837	9.7E-05	0.045	1.3E+08	7.6E+07	41	1
	0.023		30588	1.1E-04	0.047	1.0E+08	6.0E+07	-28	1
	0.024		26000	1.2E-04	0.051	7.8E+07	4.6E+07	-91	3
	0.025		23281	1.4E-04	0.053	6.3E+07	3.8E+07	-129	4
	0.026		21072	1.5E-04	0.056	5.1E+07	3.1E+07	-159	5
	0.027		19712	1.7E-04	0.059	4.4E+07	2.7E+07	-178	6
	0.028		18693	1.8E-04	0.062	3.7E+07	2.3E+07	-194	6
	0.029		18183	2.0E-04	0.065	3.3E+07	2.1E+07	-205	7
	0.030		17673	2.2E-04	0.068	2.9E+07	1.9E+07	-215	7
	0.031		17503	2.4E-04	0.071	2.7E+07	1.7E+07	-221	7

**Shot 8**									
	r (m)	***I_{max} (Amps)***	***I (Amps)***	***time (s)***	***R (m)***	***Q_{max} (W/m^2)***	***Q (W/m^2)***	***T (°C)***	***error in T (°C)***
Uncertainty		***± 100 Amps***	***± 100 Amps***	***± 0.5E-06 s***	***<1.6%***	***<0.04%***	***<0.04%***		
	0.001	105000	0	0.0E+00	0.000	N/A	N/A	N/A	N/A
	0.002		28153	1.6E-06	0.006	6.5E+09	6.1E+09	26804	866
	0.003		35607	2.3E-06	0.007	5.8E+09	4.7E+09	20872	674
	0.004		43059	2.8E-06	0.008	5.6E+09	3.9E+09	17227	556
	0.005		51191	3.6E-06	0.009	5.2E+09	3.2E+09	13904	449
	0.006		57970	4.5E-06	0.010	4.7E+09	2.5E+09	11093	358
	0.007		64747	5.2E-06	0.010	4.5E+09	2.1E+09	9190	297
	0.008		72208	6.5E-06	0.012	4.0E+09	1.8E+09	7601	246
	0.009		79669	7.8E-06	0.013	3.7E+09	1.5E+09	6461	209
	0.010		86457	9.5E-06	0.014	3.3E+09	1.3E+09	5515	178
	0.011		91887	1.1E-05	0.015	3.1E+09	1.1E+09	4715	152
	0.012		98684	1.3E-05	0.017	2.7E+09	1.0E+09	4152	134
	0.013		102774	1.6E-05	0.018	2.4E+09	8.7E+08	3596	116
	0.014		105522	1.9E-05	0.020	2.0E+09	7.6E+08	3106	100
	0.015		103559	2.6E-05	0.023	1.5E+09	6.4E+08	2546	82
	0.016		98874	3.0E-05	0.025	1.2E+09	5.3E+08	2055	66
	0.017		93506	3.5E-05	0.027	9.9E+08	4.4E+08	1656	53
	0.018		88816	3.9E-05	0.029	8.3E+08	3.7E+08	1348	44
	0.019		82772	4.3E-05	0.030	7.0E+08	3.1E+08	1072	35
	0.020		78750	4.7E-05	0.031	6.1E+08	2.6E+08	877	28
	0.021		74052	5.0E-05	0.032	5.4E+08	2.2E+08	703	23
	0.022		66000	5.7E-05	0.034	4.2E+08	1.8E+08	509	16
	0.023		58645	6.5E-05	0.037	3.3E+08	1.5E+08	351	11
	0.024		52623	7.1E-05	0.039	2.7E+08	1.2E+08	234	8
	0.025		43957	8.3E-05	0.042	1.9E+08	9.0E+07	104	3
	0.026		38653	9.3E-05	0.044	1.5E+08	7.2E+07	26	1
	0.027		34025	1.0E-04	0.046	1.2E+08	5.9E+07	-36	1
	0.028		31414	1.1E-04	0.048	1.0E+08	5.0E+07	-74	2
	0.029		29482	1.2E-04	0.050	8.9E+07	4.4E+07	-103	3
	0.030		26248	1.3E-04	0.053	7.2E+07	3.6E+07	-138	4
	0.031		24339	1.4E-04	0.055	6.1E+07	3.1E+07	-160	5

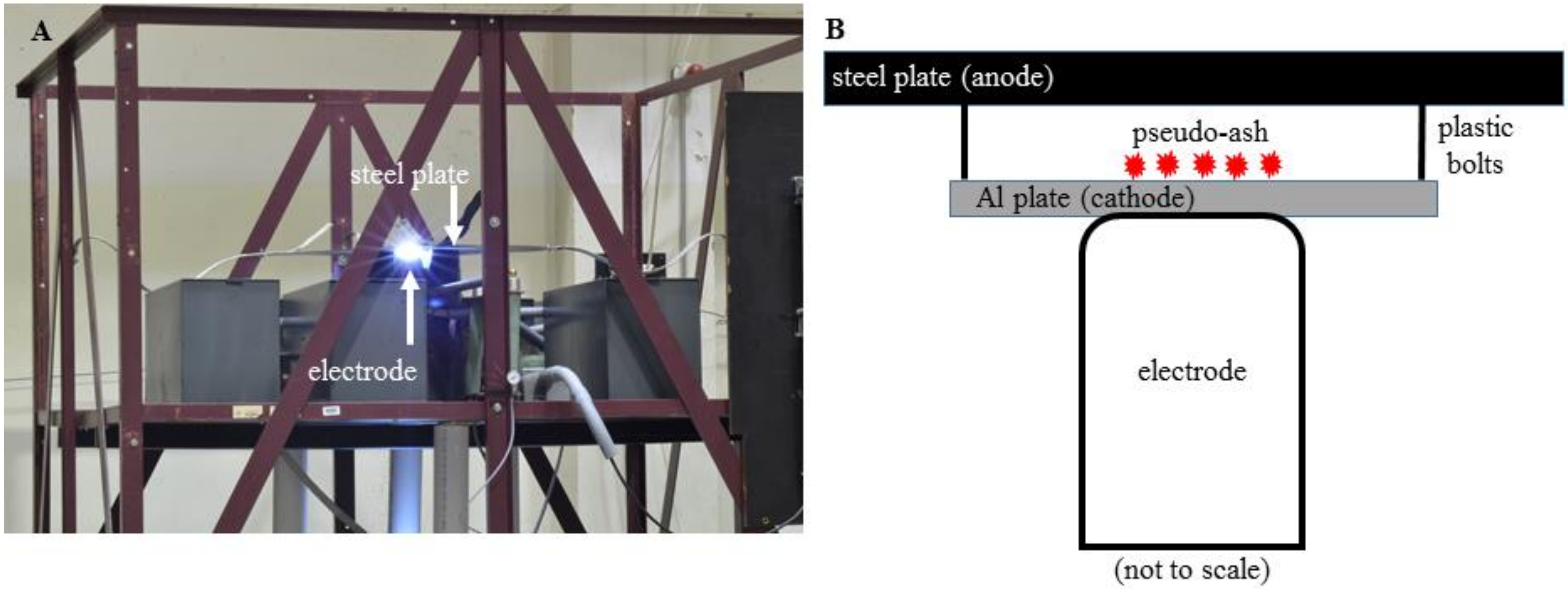

Figure S2. **High-current impulse experimental apparatus.** (**A**) Cage containing the apparatus used to conduct the high-current impulse experiments; and (**B**) schematic of instrument setup (not to scale) showing position of Al alloy plate on top of electrode. At the point of discharge, the Al plate is in direct contact with the cathode, and current arcs through this plate to the steel plate (anode) above, which is connected to ground, allowing the current to move through the pseudo-ash samples lightly dusted on top of the Al plate.
